# Differences in Somatosensory Function Related to Hand Dominance: Results of a Quantitative Sensory Testing Study in Healthy Volunteers

**DOI:** 10.2147/JPR.S470981

**Published:** 2024-09-05

**Authors:** Donna L Kennedy, Imogen Pateman, Andrew S C Rice, Caroline M Alexander

**Affiliations:** 1 Therapy Department, Imperial College Healthcare NHS Trust, London, UK; 2 Human Performance Group, Department of Surgery and Cancer, Imperial College London, London, UK; 3 Pain Research Group, Department of Surgery and Cancer, Faculty Medicine, Imperial College London, London, UK

**Keywords:** thermoreception, mechanoreception, vibration, pain, evaluation

## Abstract

**Purpose:**

Quantitative sensory testing commonly utilizes the unaffected, contralateral side as a control to detect somatosensory dysfunction. There is scant evidence that somatosensory function for the volar dominant and non-dominant hands is equivalent, therefore intra-patient comparisons are unwarranted. This study aimed to identify dominance-related differences in palmar hand somatosensation, thereby determining if the unaffected contralateral hand is a valid comparator in clinical populations.

**Participants and Methods:**

With ethical approval (IREC_13_1_10) and informed consent, 110 healthy adult volunteers’ participated in this clinical measurement study. Somatosensory function was assessed with the German Research Network on Neuropathic Pain (DFNS) quantitative sensory testing (QST) protocol. Half of the participants were tested on the dominant hand. Thirteen parameters of thermal and mechanical detection and pain threshold were evaluated at both the dorsal and volar hand (distal middle finger). Tests were performed in the same order and instructions were read from a standardized script. Results for dorsal hand tests were compared to DFNS normative data to confirm participants met study inclusion criteria. Between-group differences for age and sex were investigated with the independent samples *t*-test and Chi-square test of independence, respectively. Group differences for dominant and non-dominant hands for all 13 continuous QST parameters were investigated with the Mann–Whitney *U*-test.

**Results:**

Data for 106 participants were included in statistical analysis. Fifty percent of participants were tested on the dominant hand [n=53]; there were no differences for age or sex between groups (dominant or non-dominant hand test group). The dominant volar hand was significantly more sensitive to vibration detection threshold than the non-dominant hand (P=0.001). There were no significant differences related to dominance for other DFNS QST measures.

**Conclusion:**

For quantitative sensory testing with the DFNS protocol in healthy cohorts, the contralateral, unaffected hand is a valid control, with the exception of vibration detection threshold.

## Introduction

Somatosensory function may be impaired by diverse pathophysiology. This includes a broad range of peripheral neuropathies (disturbance of function or pathological change in a peripheral nerve^1^), including traumatic nerve lesion, nerve compression or secondary to clinical conditions such as diabetic neuropathy. Additionally, peripheral and central sensitization may result in somatosensory dysfunction.^2^ Robust sensory evaluation can aid in ascertaining the level or severity of a nerve lesion, improvement or deterioration of sensory function over time and supports differential diagnosis of the underlying causative condition.

Somatosensory function refers to the ability to interpret bodily sensations, including touch, pressure, vibration, temperature and nociception. At the periphery, cutaneous mechanoreceptors, nociceptors and thermoreceptors differ in the stimulus or modality they encode, their structure and their location in the skin, thereby generating a vast array of precise information that subserves function (Table 1). For clarity, nociception refers to the neural process of encoding noxious stimuli and does not necessarily imply that a sensation is painful.^3^ Pain, in contrast, is defined as *an unpleasant sensory and emotional experience associated with actual or potential tissue damage, or described in terms of such damage;*^4^ the distinction between the phenomena is an important one.

**Table 1:** Classification of Somatosensory Receptors

Receptor	Modality	Axon
**Mechanoreceptors**		
Meissner corpuscles	Fine discriminative touch	Aβ
Merkel discs	Light touch; superficial pressure	
Hair follicle receptors	Light touch (hairy skin)	
Ruffini endings	Continuous touch; pressure	
Pacinian corpuscles	Vibration	
**Nociceptors**		
Free nerve endings	Sharp pain (Pin prick)	Aδ
	Thermal- cold	
	Dull aching pain	C
	Thermal - heat	
**Thermoreceptors**		
Free nerve endings	Cold detection	Aδ
	Warm detection	C

**Notes**: Adapted from: Brodal;^37^ Nolte;^38^ Young, Young, Tolbert.^39^

Clinicians have long sought to evaluate, or quantify, somatosensory function in clinical cohorts. Quantitative sensory testing (QST) describes the measurement of sensory loss and sensory gain (allodynia; hyperalgesia) in response to graded multi-modal stimuli. Sensory testing is considered “quantitative” where the intensity of the stimulus and/or the participants’ response, is measurable.^5^ QST is a psychophysical measure as it is dependent on the understanding, active participation and cooperation of the subject, in contrast to objective measures such as nerve conduction studies which are independent of the subjects’ participation.

In the 1950s, QST pioneer, neuroscientist Sidney Weinstein, collaborated with Josephine Semmes to develop the Semmes-Weinstein Pressure Aesthesiometer, initially for the evaluation of sensory disturbance secondary to brain injury.^6^ Semmes-Weinstein monofilaments and the test’s successor, the Weinstein Enhanced Sensory Test (WEST), are handheld sensory tests that use nylon filaments of variable diameters to measure touch pressure or mechanical detection. The monofilament is applied to the skin until it bends, thereby controlling for and consistently delivering the same force.^7^ Since their development, Semmes-Weinstein monofilaments have demonstrated clinical utility and reliability for the evaluation of peripheral neuropathy and traumatic nerve injury^8^ and are widely used in clinical practice. Further innovation in QST was seen in 1978 when plastic surgeon and founder of modern peripheral nerve injury science, A. Lee Dellon, reported on the clinical utility of the moving two-point discrimination test^9^ and subsequently in 1980 on the use of vibration to evaluate peripheral nerve injury.^10^

To date, QST in the clinical setting has focused predominantly on such aforementioned tests of large fiber mechanoreception; rarely are small fiber nociception and thermoreception tests described in clinical recommendations.^11^ However, the complexity of sensibility means a battery of tests, including those for small fiber nociception and thermoreception, best inform a comprehensive evaluation of function. Historically, differences in QST equipment and testing protocols precluded the synthesis of sensory testing evidence. Therefore, to improve standardisation and promote interpretability, the German Research Network on Neuropathic Pain (DFNS) published a comprehensive protocol for QST.

The DFNS QST protocol comprises thirteen measures of small (Aδ & C) and large fiber (Aβ) sensory function and pain sensitivity, resulting in a thorough appraisal of somatosensory function.^12^ The DFNS QST protocol has been implemented to define reference values in healthy subjects for the face, dorsal foot, dorsal hand^13^,^14^ and the trunk.^15^ Importantly, the precision afforded by the protocol has demonstrated that disturbances in somatosensory function vary between neuropathic conditions and furthermore between patients with the same clinical condition.^16^

Normative data for DFNS QST demonstrate there are no differences in sensory parameter sensitivity between the left and right sides of the face, dorsal hand and dorsal foot.^14^ However, such differences have not been explored for the volar, dexterous surface of the hand. With other QST protocols, the limited, low quality published data on differences in large fiber somatosensory function related to dominance are conflicting and warrant exploration.^17^,^18^ To date, there is no published evidence on small fiber somatosensory differences at the palmar hand related to dominance.

The bilateral test sites compared with the DFNS QST protocol (face, dorsal foot, dorsal hand, trunk) are not prehensile dermal surfaces; therefore, it is not expected that sensitivity may be affected by function or exposure over time. In contrast, the palmer fingertips of the dominant hand perform fine dexterity tasks, such as manipulating small objects, therefore this might offer a learning or training benefit, thereby increasing sensitivity. Conversely, the dominant hand is exposed to greater mechanical and thermal stimuli through the performance of everyday tasks, and as a result may be less sensitive. As differences in DFNS QST parameters between the palmar dominant and non-dominant hands have not been explored, it is unclear if the contralateral palmar hand is a robust comparator. Therefore, this study aimed to explore differences between the dominant and non-dominant palmer hand surface for large fiber and small fiber somatosensory function using the DFNS QST.

### Hypothesis

In healthy adult volunteers, the palmar surface of the dominant hand will be more sensitive to mechanical and thermal stimuli than the non-dominant hand.

### Study Aims

- To determine if the dominant hand is more sensitive to mechanical and thermal stimuli than the non-dominant hand.
- Evaluate the clinical practicality of the DFNS QST measures for routine clinical practice.

## Methods

This study was conducted in accordance with the Declaration of Helsinki, and all applicable local regulations. Ethical approval (IREC_13_1_10) was received from the Imperial College Research Ethics Committee on May 13th, 2014, for Healthy Volunteer Quantitative Sensory Testing (QST): a Quality Control Study. This manuscript reports a secondary analysis of an existing data set and is based on the thesis of DLK.^19^,^20^

### Sample Size

This study is a secondary analysis of an existing dataset; all data of that existing set was used for this study.^19^ Relevant data on the effects of hand dominance on sensory function^9^,^17^,^18^ is lacking, and there are no published data for DFNS QST testing at the palmar hand surface to support a sample size calculation. Therefore, sample size for this study was estimated pragmatically at N=100 (50 per group; dominant versus non-dominant hand), based on the DFNS published reference data for other body sites.^14^

### Recruitment

Recruitment began on July 30th 2014 and concluded on July 7th 2017. Healthy volunteers were recruited from the staff, students and visitors to Imperial College Healthcare NHS Trust, Chelsea & Westminster Foundation Trust Hospital and Imperial College London by study posters. Participants volunteered their time and were not reimbursed for time or travel. Recruitment strategy was to enroll equal numbers of males and females between 18 and 80 years of age and to test equal numbers of dominant and non-dominant hands to enable hand dominance comparisons. While testing both the dominant and non-dominant hand in the same participant (intra-subject comparison) is desirable, this approach was rejected for the significant increase in testing time that we felt would impede recruitment. Tests were completed at Chelsea and Westminster Foundation Trust Hospital and at Charing Cross Hospital.

### Participants

Participants included healthy adults 18 years or older with adequate English language for comprehension of the study and to give informed consent. In keeping with consensus group recommendations for the validation of volunteers as “healthy” in QST studies,^21^ participants completed a health screening questionnaire to ensure they met the study inclusion criteria and provided informed, written consent. Those with a history of potentially confounding conditions, including previous trauma to the limbs, rheumatoid arthritis, renal failure, peripheral neuropathy or cervical radiculopathy, pregnancy and those taking pain medication were excluded. Additionally, post-testing, QST results for participants’ dorsal hand were compared to published DFNS reference data.^14^ Data for those with more than one abnormal measure were omitted from study analysis to exclude those with a potentially undiagnosed and/or subclinical neuropathy. Excluded participants were not informed, given the experimental rather than diagnostic nature of the tests under investigation.

### Procedure

The first enrolled participant was tested on the dominant hand and thereafter, testing in subsequent participants alternated between non-dominant and dominant hands to ensure equal numbers for comparison. Participants underwent the DFNS QST battery^12^ at the dorsal hand (radial nerve innervation) and volar distal middle finger (median nerve innervation), completed at one session. Test results were manually recorded on the DFNS QST documentation form.

All QST equipment at both study sites was newly purchased or calibrated prior to study commencement. The standardized battery of 7 tests measuring 13 parameters^12^ was conducted in the same order, using standardized instructions. Participants were seated in a quiet room with the test hand supported on a table. Participants first received instructions; the tests were then practiced at an adjacent area before the test was conducted on the dorsal hand followed by the volar hand. All palmar hand measures were performed at the volar distal middle finger except pressure pain threshold which was tested at the thenar eminence.

Details of the QST testing procedure have been previously reported.^19^,^22^ In brief, thermal testing used a calibrated MSA thermal sensory analyzer (Somedic, Sweden) with a 25×50 mm Somedic thermode for dorsal hand tests and an 18 mm^2^ metal Somedic thermode for volar middle finger tests. Thermal thresholds were tested using a baseline temperature of 32°C and ramping at 1°C/s with limits of 5°C and 50°C. Mechanical detection threshold was tested using glass monofilaments (Optihair2-Set, Marstock Nervtest, Germany) with bending forces between 0.25 and 512 mN. Blunt probes with forces ranging from 8 to 512 mN (pinprick stimulator, MRC, Heidelberg, Germany) were used to test mechanical pain threshold (MPT), mechanical pain sensitivity (MPS), and wind-up ratio. Dynamic mechanical allodynia was tested with a cotton wisp, a cotton bud (Q-Tip), and a soft brush (Somedic, Sweden). Vibration detection threshold testing used a Rydel–Seiffer graded tuning fork (64 Hz, 8/8 scale).^19^ Pressure pain threshold was tested with a pressure algometer (FDN100; Wagner Instruments, Greenwich, CT) with a surface area of 1 cm2 and by applying pressure at a rate of 1 kg/cm2 per second.

### Statistical Analysis

Data for included participants were entered into IBM SPSS v 24 and analyzed for distribution properties with the Kolmogorov–Smirnov test and by visual inspection of histograms. Participant characteristics were summarized using descriptive statistics. Differences in continuous measures for the dominant and non-dominant hand were investigated with the independent samples *t*-test for normally distributed measures and the Mann–Whitney *U*-test for non-parametric measures. QST results for dorsal hand tests were compared to the DFNS published age and gender-specific normative values.^13^ Results below the 2.5 centile and above the 97.5 centile, ie results outside the 95% reference range, were considered abnormal. Because 95% of QST normative data lie within two standard deviations of the mean values, it can be expected that up to 5% of healthy volunteers will present with at least one abnormal test.^14^ Therefore, data for participants with more than one abnormal dorsal hand QST value were excluded.

## Results

### Participants

**Figure 1:**
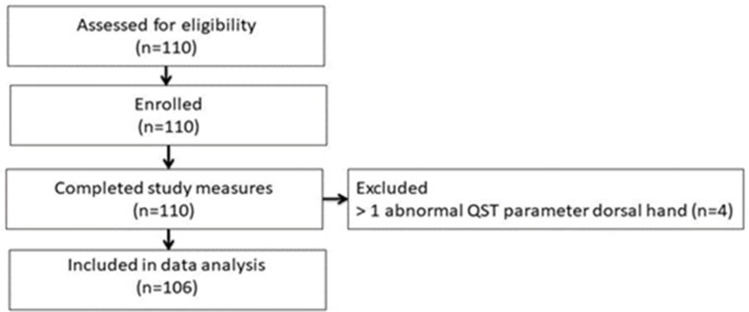
Recruitment and enrolment flow diagram.

One hundred and ten healthy volunteers who gave informed consent participated in the study. Results for four participants were excluded from analysis for having more than one abnormal test value at the dorsal hand (Figure 1). Data for 106 participants [66 females (62%), age mean (standard deviation) 42.2 (15.8)] was included for analysis. Ninety-five (90%) participants reported to be right hand dominant. Testing was divided evenly with fifty percent of tests performed on the participant’s dominant hand (dominant hand group [n=53]), fifty percent on the non-dominant hand (non-dominant hand group [n=53]). The dominant hand and non-dominant hand groups had a mean (standard deviation) age of 42.3 (15.5) and 42.1 (16.2) years respectively; the groups were not significantly different for age (p=0.5). The dominant hand group contained females n=32; males n=21 and the non-dominant hand group females n=34; males n=19; a chi-square test of independence identified no significant difference between groups for sex (p=0.84) (Table 2).

**Table 2:** Baseline Characteristics for Sample by Test Site

	Dominant Hand (n=53)	Non-Dominant Hand (n=53)	Sig
Age			
Mean years (sd)	42.28 (15.5)	42.09 (16.2)	0.5
Sex			
Male n=	21	19	0.84
Female n=	32	34	

**Abbreviations**: Sd, standard deviation; sig, statistical significance.

### Between Hand-Dominance Analysis

**Figure 2:**
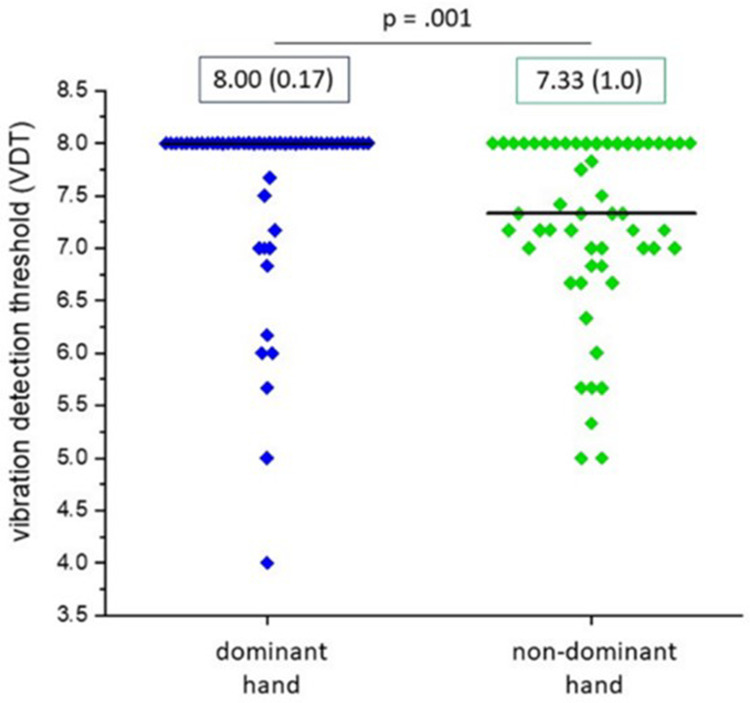
Distribution (median [interquartile range]) of scores for vibration detection threshold (VDT) for the sample, comparing dominant (blue) and non-dominant (green) hand groups. Each participant is represented by a diamond, solid black line represents the median. VDT is the mean disappearance threshold of 3 trials measured on a 0 to 8 scale to the closest half-point; 8.0 represents greatest sensitivity.

Results by QST parameter for the dominant and non-dominant hand groups are reported in Table 3. As anticipated, participants did not demonstrate paradoxical heat sensations or dynamic mechanical allodynia, measures of pathological function; therefore, these measures are not reported. The between-group analysis indicated that the dominant hand was significantly more sensitive to vibration detection threshold than the non-dominant hand (p=0.001) (Figure 2). No significant differences in somatosensory function for dominant compared to non-dominant hands were found for the remaining QST parameters.

**Table 3:** Comparison of QST Results for the Dominant and Non-Dominant Hand Tested at the Volar, Distal Middle Finger in Healthy Participants

Parameter	Dominant Hand	Non-Dominant Hand	*p value*
	*Median [95% CI]*	*Median [95% CI]*	
Cold detection threshold (°C)	3.23 [2.47, 4.07]	2.73 [2.23, 3.4]	0.57
Warm detection threshold (°C)	4.73 [3.47, 5.7]	4.17 [3.17, 6.47]	0.59
Thermal sensory limen (°C)	8.13 [7.17, 10.7]	8.4 [6.73, 10.57]	0.92
Cold pain threshold (°C)	10.1 [5.6, 15.1]	11.2 [8.43, 15.83]	0.5
Heat pain threshold (°C)	46.9 [45.5, 47.73]	46.43 [45.3, 48.17]	0.76
Mechanical detection threshold (mN)	0.18 [0.18, 0.25]	0.18 [0.18, 0.18]	0.36
Mechanical pain threshold (mN)	128 [90.51, 181.02]	111.43 [90.51, 157.59]	0.79
Mechanical pain sensitivity (0–100)	0.32 [0.23, 0.53]	0.34 [0.27, 0.49]	0.82
Wind up ratio	1.8 [1.47, 2.22]	2.24 [1.8, 2.67]	0.22
Vibration detection threshold (x/8)	8 [8.0, 8.0]	7.33 [7.0, 8.0]	**0.001**
Pressure pain threshold (k/cm^2^)	422 [386, 468]	383 [334, 461]	0.1

**Notes**: Cold and warm detection threshold and cold and heat pain threshold are calculated as the change in °C from 32 °C baseline. 95% CI; 95% confidence interval (lower bound, upper bound). Statistically significant results in bold.

### Clinical Practicality of DFNS QST Protocol for Routine Clinical Practice

Clinical practicality was assessed in keeping with the evaluation of administrative and respondent burden.^23^,^24^ The standardized equipment for DFNS QST testing is expensive, and potentially beyond the available resources of many clinical settings.^12^ The standardized equipment must be handled with care to avoid damage; however, over the course of this study there were no equipment failures or breakages that precluded testing. Testing requires on average thirty minutes for each battery of thirteen tests. It is recommended that DFNS QST is completed by a trained investigator; training therefore adds to the overall cost and time required. Testing should be conducted in a quiet, temperature-controlled room; this may not be practical in some clinical environments or geographical regions.

Regarding respondent burden, it is recommended that a test place no undue physical or emotional strain on respondents.^24^ For patients or participants undergoing DFNS QST testing, time is required for travel to the testing site as well as for completion of the test. Patients require an adequate level of verbal English comprehension as required to follow standardized test instructions. No special requirements are placed on respondents prior to testing, such as modification to medication or meals. Regarding participant acceptability of the test battery, there were no dropouts during testing, ie no participant refused to continue the test battery to completion and there were no adverse events secondary to testing.

## Discussion

The aim of this novel study was to determine if there are differences in sensitivity between the dominant and non-dominant hands in response to mechanical and thermal stimuli, as tested with the DFNS quantitative sensory testing protocol.^12^ Secondarily, we aimed to consider the clinical practicality of the DFNS QST measures for routine practice. Relative (between-group) differences in somatosensory function were explored, rather than absolute differences (intra-individual) to ensure adequate study participation. This study demonstrated that for tests at the volar, distal middle finger (median nerve distribution), with the exception of vibration detection threshold, there is no difference in somatosensory function for the dominant as compared to non-dominant hand. Symmetry in sensitivity between sides of the body in healthy volunteers is consistent with findings of the DFNS, where for the face, dorsal hand and dorsal foot there are no differences for tests of the left and right side of the body^14^ and therefore the unaffected hand can be used as a comparator for sensory testing. An important caveat, however, is relevant to the testing of somatosensory function in those with central sensitization and chronic pain, whereby there may be effects in the contralateral limb which preclude its use as a control.^25^

Existing literature on somatosensory differences between the dominant and non-dominant hand is sparse, particularly regarding small fibre innervated somatosensory function. In a cross-sectional study by Hage et al^17^ involving 130 healthy participants, a Semmes-Weinstein Pressure Aesthesiometer was used to measure mechanical detection threshold on the pad of the index finger of both hands in each subject. In concurrence with our results, they reported no difference between the dominant and non-dominant hand with regards to mechanical detection threshold. In contrast to Hage et al^17^ and our findings, Ozcan et al’s^26^ cross-sectional study involving 60 healthy volunteers found that right-handed participants had significantly higher pain pressure thresholds in the dominant hand compared to the non-dominant hand when using a dolorimeter to assess pressure pain threshold at the fingertip of the index finger. Nonetheless, no difference was found for those who were left-handed, and the authors concluded more research is required to explore differences in sensory function between the dominant and non-dominant hands in larger cohorts and with more detailed experiments.^26^

The finding that vibration detection threshold, in contrast to results for other sensory parameters, differs between the dominant and non-dominant median nerve innervated volar hand, was unexpected and counter intuitive. The volar, dominant fingertips perform dexterous tasks requiring great sensitivity and precision while also being exposed to deep pressure and ranges of thermal stimuli; therefore, it was hypothesized that the dominant hand would demonstrate greater sensory sensitivity across testing parameters.

The explanation for a difference in vibration detection threshold related to hand dominance is largely unknown. Vibration detection threshold is a large fiber (Aβ) mediated measure of mechanoreception. Vibrations are oscillations that occur around an equilibrium point; they occur all around us in daily life.^27^ Historically, the perception of vibrations was thought to be crucial to survival, such as for detecting predator movement.^27^ However, in modern times, the original advantages are less essential for survival. Nonetheless, a decrease in vibration detection threshold is associated with the presence of various spinal cord diseases and may be an early sign of peripheral neuropathy.^28^

In this sample of healthy participants, large fiber mechanoreception was also evaluated with a test of mechanical detection threshold with no differences for dominance detected. This perhaps implicates differences in Pacinian corpuscle function, the receptor organ for vibration detection, in dominance-related differences in vibration detection. Pacini corpuscles are known to be clustered in the fingertips and are crucial for proprioception.^29^ Perhaps greater sensitivity to vibration detection threshold in the dominant hand is associated with greater proprioception related to functional demands.^29^ Nevertheless, it must be considered that the observed difference in vibration detection threshold may be due to an anomaly in the sample of healthy volunteers and not representative of the larger population.

**Figure 3:**
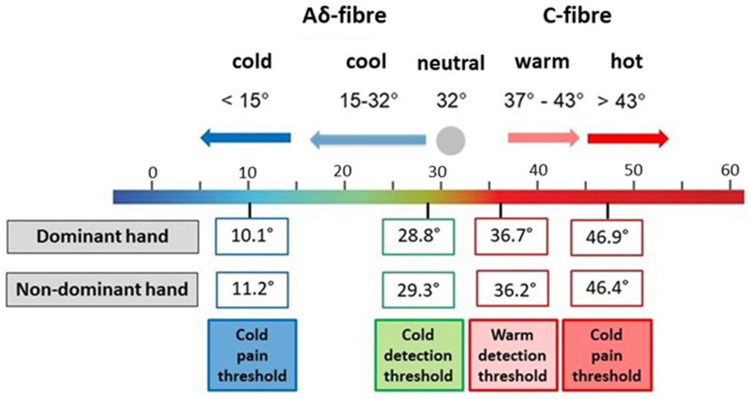
Results of thermal testing in healthy volunteers. Thermal detection and pain thresholds illustrated as median values in °C. An individual’s detection and threshold values are calculated as the change from 32°C.

The results of this study have important implications for clinical practice. In this study, thermal testing (Figure 3) demonstrates that cold and warm detection at the volar fingertips is a precise sensory function, with a change of between 3°C and 4°C from 32°C perceived as cooling or warming, respectively. This precision in thermal perception is in keeping with the DFNS results for other body sites.^14^ For the clinical assessment of thermal detection using more crude methods such as water filled test tubes, the results reported here may be extrapolated to determine relevant test temperatures. In this study, thermal tests were conducted using an 18 mm^2^ metal Somedic thermode. However, thermal perception is faster, and more precise, when tested over a greater surface area or for longer exposures,^30^,^31^ therefore testing parameters used in clinical practice may impact results and warrant consideration.

**Figure 4:**
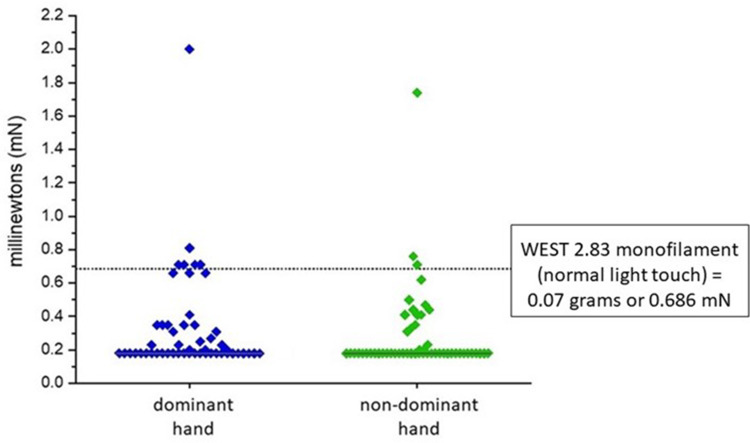
Results of mechanical detection threshold testing in healthy volunteers. Dominant hand tests in blue, non-dominant in green. Each participant is represented by a diamond. The solid line represents the median. The dotted line at 0.686 mN is the equivalent of 0.07 grams, or the monofilament with the lightest bending force in the WEST assessment tool.

Mechanical detection threshold, or static touch detection, is a large fiber mediated measure of mechanoreception and is commonly assessed in the clinical setting with Semmes-Weinstein monofilaments^6^ or the Weinstein Enhanced Sensory Test (WEST). The WEST is a modified version of Semmes Weinstein monofilaments and reportedly demonstrates improvements in instrument design and calibration,^32^ however the tools have the same testing thresholds.^6^ The WEST contains five calibrated monofilaments, the lightest having a bending force of 0.07 grams (or 0.686 mN), considered the upper threshold of normal light touch.^7^ In the current study, mechanical detection threshold was evaluated with glass Marstock monofilaments. Median mechanical detection threshold was 0.18 mN for both dominant and non-dominant hands (Figure 4). Importantly, our results reveal a floor effect in the WEST mechanical detection test, whereby most healthy participants demonstrate greater mechanical detection sensitivity at the volar fingertips than is identifiable with the WEST. This suggests that when using the WEST in clinical assessment, a patient may have loss of mechanical detection undetectable given the sensitivity of the tool, and therefore correlation of testing with patient reported symptoms is essential.

While the DFNS QST protocol affords standardisation and precision, the cost of equipment and maintenance and time required for training and testing may reduce its utility for clinical settings. The comprehensive battery of 13 tests requires thirty minutes per test site, which may place it out of scope for routine care. In practice, clinicians have traditionally used readily available, low-cost tools for sensory evaluation; test tubes filled with hot and cold water have been recommended to assess warm and cold detection and heat pain threshold,^11^ ice for cold pain threshold^33^ and toothpicks for mechanical pain threshold and wind up ratio.^34^ An eraser or the clinician’s thumb can be used to assess pressure pain threshold and tuning forks to assess vibration.^11^ While being inexpensive and quick to perform, these crude tests nonetheless lack precision, are generally not quantifiable and the ability to detect small but clinically important changes in function is impeded. The demand for a compromise between precision and clinical utility in quantitative sensory testing has led to the more recent development of “bedside QST” protocols. Thus far, reliability data for these low‐cost and time-efficient bedside QST batteries suggest they may have clinical utility in the healthcare setting.^34–36^

### Study Strengths and Limitations

Several measures were taken to reduce study bias. Due to the absence of relevant data from previous studies investigating the effect of hand dominance on sensory function, power calculations could not be utilized to calculate a desired sample size prior to recruitment. However, post-hoc testing indicated that the sample size recruited was more than sufficient to detect a significant difference between groups. The definition of “healthy” is often variable across QST studies;^21^ therefore, the screening protocol for this study was based on the EUROPAIN and NEUROPAIN consensus statement.^21^ This two-level approach, using standardized questions to collect information on current health status, past medical history and the volunteer’s motivations to enroll, aimed to reduce the variability of results and reduce the likelihood of false negatives and false positives. To reduce the risk of common method bias, a strict testing protocol, including reading standardized instructions, was implemented to avoid variance in methodology between participants. As QST is a psychophysical measure, it is paramount that the testing environment is free of distraction. Therefore, all testing was carried out in a quiet environment. QST was also practiced on an adjacent area, prior to testing the median nerve, to ensure that participants fully understood the process.

In this study, all QST measures were completed by one investigator, thereby increasing the potential for systematic error in the study findings. Comparisons of somatosensory function for dominant and non-dominant hands were relative; half the sample was tested on the dominant hand and half the non-dominant hand, in contrast to absolute differences where both hands were tested in the same subject. This decision was taken for pragmatic reasons. Adding an additional test site would have increased testing time by thirty minutes for each participant and is likely to have had an adverse impact on recruitment. It would be of interest to further explore intra-participant differences in volar hand somatosensation related to hand dominance.

Consistent with results for QST in healthy volunteers reported by the DFNS (Rolke et al, 2006a), data for this study were not normally distributed. This is as anticipated in a healthy population secondary both to testing parameters and due to floor and ceiling effects that result from limitations in the precision and scale of measurement in psychophysical measures such as QST. For example, temperature limits (5°C; 50°C) are imposed in thermal tests both to avoid the risk of tissue damage for the participant and the possibility of damage to the thermode. As a result, a floor effect of 5°C is seen in the assessment of cold pain threshold and a ceiling effect of 50°C for heat pain threshold. The presence of floor and ceiling effects suggests the normal distribution of scores would include scores beyond the range of scores available in the instrument, and therefore sensitivity is lost. Similarly, for mechanical detection threshold and vibration detection threshold, scores in healthy volunteers are skewed because many participants can perceive the lightest or smallest stimulus available within the tool range.

## Conclusion

For the evaluation of somatosensory function with the DFNS QST protocol in adult healthy volunteers at the volar median nerve innervated hand, the dominant hand is significantly more sensitive than the non-dominant hand to vibration detection threshold. For DFNS QST tests of thermal and mechanical detection and pain, there is no difference in function related to hand dominance. The unaffected, contralateral hand can be used as a robust comparator for sensory testing in healthy volunteers. Further research is required to determine if the contralateral hand can be used as a robust comparator in those with unilateral limb pain.

## Data Availability

Datasets used and/or analysed during the current study are available from the corresponding author on reasonable request.

## Abbreviations

DFNS, German Research Network on Neuropathic Pain; QST, Quantitative Sensory Testing; VDT, Vibration detection threshold; WEST, Weinstein Enhanced Sensory Test.
